# ELECTRONIC MEDICAL RECORD ALGORITHM TO IDENTIFY UNDIAGNOSED (PRE)DIABETES: A CASE STUDY FROM HAWAIʻI

**DOI:** 10.1093/geroni/igz038.3397

**Published:** 2019-11-08

**Authors:** David Stupplebeen, Tetine L Sentell, Lance Ching, Blythe Nett, Hermina Taylor, Tiffany Lemmen, Catherine M Pirkle

**Affiliations:** 1 University of Hawaii at Manoa, Honolulu, Hawaii, United States; 2 Office of Public Health Studies, University of Hawaii at Manoa, Honolulu, Hawaii, United States; 3 Hawaii Department of Health, SEEO, Honolulu, Hawaii, United States; 4 Hawaii Department of Health, Honolulu, Hawaii, United States; 5 The Queen's Health System, Honolulu, Hawaii, United States

## Abstract

An estimated one-quarter of United States’ older adults (≥65 years) have diabetes (DM) while half have prediabetes (PreDM). Timely diagnosis can prevent disease progression, but significant proportions of PreDM/DM are undiagnosed. Among Hawai‘i adults, one-third of diabetes and two-thirds of prediabetes cases are undiagnosed; rates for older adults are unknown. Algorithms integrated into Electronic Medical Records (EMR) may improve care by identifying probable undiagnosed cases in patient panels using clinical/laboratory measures. We assessed one algorithm developed by the Hawai‘i Department of Health that identified individuals overdue for screening or with Pre/DM using the records of 20,362 adult patients (51.33% were >65) from a major state health system. 6,371 (31.3%) patients were excluded from analysis; they had no HbA1c screening in the past year or were overdue for screening (70%) based on standard guidelines. Of the remaining 13,991 patients, 7317 were older adults; 6130 (84%) had a PreDM (50.6%) or DM (33.2%) HbA1c value; the rest were controlled or false-positive. Of those older adults with probable PreDM/DM, 38.6% were undiagnosed. Adults >65 were significantly more likely to be flagged with undiagnosed PreDM compared to their younger counterparts (58 versus 54%, p<.001). Notably, 61% of older men flagged with PreDM were undiagnosed. Of the 5,737 patients identified with DM, 22% of those 65 were undiagnosed. Given the recognized high burden of diabetes among older adults, results indicate substantial missed opportunities for the prevention and early treatment of this condition as identified by an EMR algorithm.

